# Data on exergy and exergy analyses of drying process of onion in a batch dryer

**DOI:** 10.1016/j.dib.2018.10.132

**Published:** 2018-10-30

**Authors:** J. Adewale Folayan, F.N. Osuolale, P.A.L. Anawe

**Affiliations:** a College of Engineering, Covenant University, OTA, Nigeria; b Department of Chemical Engineering, LAUTECH, Ogbomoso, Nigeria

**Keywords:** Exergy, Exergetic sustainability index, Exergetic improvement potential, Onion, Cabinet dryer

## Abstract

Today׳s engineering systems and machine are so sophisticated that mere energy analysis cannot accurately and reliably describe the thermodynamic behaviour, viz-a-viz the energy changes occurring in these complex systems. Hence, a more efficient and realistic parameter that provides us with useful information about thermodynamic losses and energy efficiency improvement potential is the exergy analyses. Fresh samples of onion fruits were washed with distilled water to remove particles and contaminants that can adversely affect the experimental results. Hence, 36.50 g of the sample at different thicknesses of 0.50 cm, 1.00 cm and 1.50 cm were taken into the cabinet dryer for drying at different temperatures of 65 °C, 75 °C,85 °C and 95 °C and the weight loss at each temperature and thickness was determined with the aid of a digital weighing balance. Hence, it was on this premise that the exergy analyses in terms of exergy loss, exergetic improvement potential and exergetic sustainability index of drying process of onion at different drying air temperatures, drying periods and thicknesses in a cabinet dryer was performed.

**Specifications table**

**Table:** 

Subject area	Chemical Engineering
More specific subject area	Thermodynamics
Type of data	Tables, figures, images,
How data was acquired	Experimental
Data format	Raw, Analyzed
Experimental factors	Energy usage optimization in drying process depends on various factors such as air temperature, feeding rates, relative humidity or wet-bulb depression, air velocity, air mass flow rate and particles size, shape and arrangement.
Experimental features	Exergy analysis in terms of exergy inflow, exergy of dried product, exergy outflow, exergy loss, exergy efficiency, exergetic sustainability index and exergetic improvement potential of drying process of onion in a cabinet dryer at different drying periods were evaluated under drying temperatures of 65 °C,75 °C,85 °C and 95 °C and particle thickness of 0.50 cm,1.00 cm and 1.50 cm.
Data source location	Nigeria.
Data accessibility	Data are available within this article
Related research article	None

**Value of the data**

• The data showed the optimum temperature condition for efficient energy usage during the drying of onion in a cabinet dryer.
• The data furnished us with reliable information as regards the energy and exergy efficiency of batch dryers used in various drying processes.
• The data examined the effect of particle size on exergy and exergy efficiency during drying processes.
• The data give us a hint on the likely sources and location of thermodynamic inefficiencies during drying process and where improvement potential is possible.
• The data will serve as guide on dryer selection for various individuals and industries involved in food stuff preservation.

## 1 Data

**Fig. 1:**
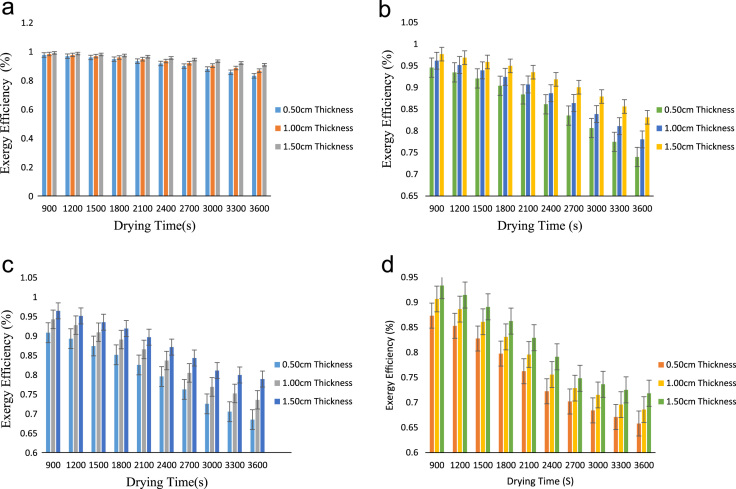
a: Exergy efficiency at 65 °C drying temperature. b: Exergy efficiency at 75 °C drying temperature. c: Exergy efficiency at 85 °C drying temperature. d: Exergy efficiency at 85 °C drying temperature.

**Fig. 2:**
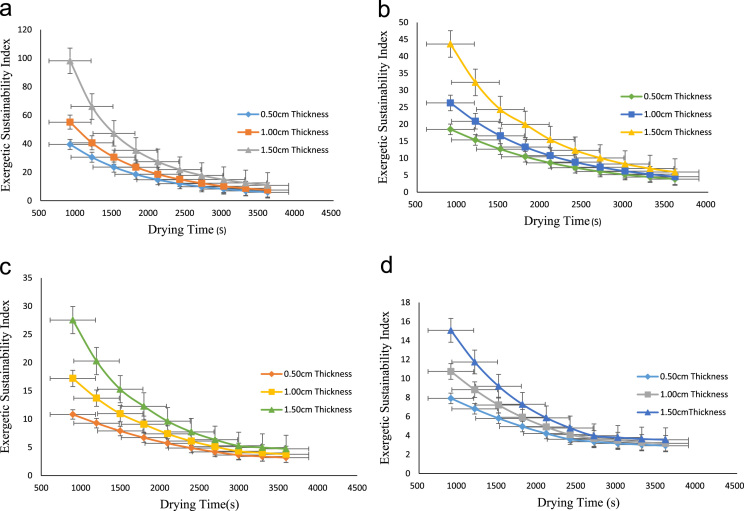
a: Exergetic sustainability index at 65 °C. b: Exergetic sustainability index at 75 °C. c: Exergetic sustainability index at 85 °C. d: Exergetic sustainability index at 95 °C.

**Fig. 3:**
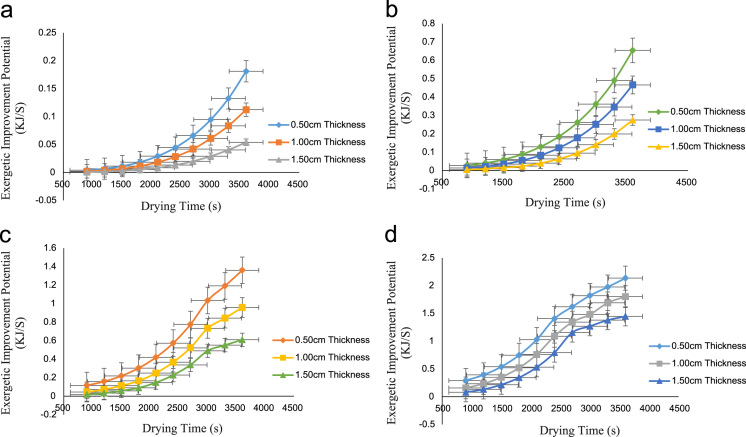
a: Exergetic improvement potential at 65 °C. b: Exergetic improvement potential at 75 °C. c: Exergetic improvement potential at 85 °C. d: Exergetic improvement potential at 95 °C.

The data obtained from this research work comes from the exergy analysis of drying process of onion in a cabinet dryer. The exergy inflow, exergy of dried product, exergy outflow, exergy loss, exergy efficiency, exergetic sustainability index and exergetic improvement potential were evaluated at various drying temperatures of 65°C,75 °C,85 °C and 95 °C and particle thickness of 0.50 cm,1.00 cm and 1.50 cm. Table 1 showed the exergy inflow at various drying air temperatures while Table 2a, Table 2b, Table 2c, Table 2d described the exergy of dried products at different temperatures and thicknesses. The exergy outflow at various drying air temperatures and thicknesses is presented in Table 3a, Table 3b, Table 3c, Table 3d and the exergy loss is showed by Table 4a, Table 4b, Table 4c, Table 4d. Similarly, the exergy efficiency of the drying process at different drying air temperatures is presented in Fig. 1**a–d** while the exergetic sustainability index at various temperatures is described by Fig. 2**a–d.** Finally, the exergetic improvement potential at different drying air temperatures is vividly presented in Fig. 3**a–d**.

**Table 1:** Exergy inflow (kJ/s) at various drying temperatures.

**Temperature (°C)**	**EX**_**air**_**(kJ/s)**	**EX**_**Fo**_**(kJ/s)**	**Ex**_**inflow**_**(kJ/s)**
65	3.7326	2.5859	6.3185
75	5.7173	3.9608	9.6781
85	8.0825	5.5938	13.6763
95	10.8052	7.4709	18.2761

**Table 2a:** Exergy of dried product (kJ/s) at 65 °C.

**Time(s)**	**0.50 cm thickness**	**1.00 cm thickness**	**1.50 cm thickness**
900	2.425892	2.471284	2.521512
1200	2.378722	2.430267	2.490297
1500	2.318074	2.378996	2.452145
1800	2.243950	2.317470	2.407056
2100	2.156348	2.245690	2.355030
2400	2.055269	2.163655	2.296068
2700	1.940713	2.071367	2.230168
3000	1.812680	1.965406	2.157332
3300	1.671170	1.859445	2.081028
3600	1.516182	1.743229	2.001255

**Table 2b:** Exergy of dried product (kJ/s) at 75 °C.

**Time(s)**	**0.50 cm Thickness**	**1.00 cm Thickness**	**1.50 cm Thickness**
900	3.437906	3.592653	3.739042
1200	3.331977	3.497583	3.661576
1500	3.197157	3.377494	3.563452
1800	3.033447	3.232387	3.475657
2100	2.840847	3.062261	3.336217
2400	2.619357	2.867117	3.176120
2700	2.368978	2.646955	3.000530
3000	2.089708	2.401774	2.793953
3300	1.781548	2.131574	2.571883
3600	1.444499	1.836356	2.329155

**Table 2c:** Exergy of dried product (kJ/s) at 85 °C.

**Time(s)**	**0.50 cm Thickness**	**1.00 cmThickness**	**1.50 cm Thickness**
900	4.330610	4.799319	5.097298
1200	4.121461	4.596531	4.919816
1500	3.863101	4.346425	4.699737
1800	3.555529	4.089560	4.479659
2100	3.198746	3.744821	4.174389
2400	2.792751	3.352764	3.826523
2700	2.337545	2.920149	3.443161
3000	1.833128	2.426698	3.003004
3300	1.556313	2.196871	2.846820
3600	1.279498	1.973804	2.704834

**Table 2d:** Exergy of dried product (kJ/s) at 95 °C.

**Time(s)**	**0.50 cm Thickness**	**1.00 cm Thickness**	**1.50 cm Thickness**
900	5.161715	5.769821	6.258066
1200	4.788759	5.398904	5.914313
1500	4.326293	4.937318	5.482418
1800	3.774318	4.385064	4.962382
2100	3.132833	3.742141	4.354203
2400	2.401839	3.008549	3.657883
2700	2.028882	2.515642	2.873422
3000	1.700681	2.266715	2.653067
3300	1.461989	1.912283	2.450341
3600	1.223297	1.730946	2.326943

**Table 3a:** Exergy outflow (kJ/s) at 65 °C.

**Time(s)**	**0.50 cm Thickness**	**1.00 cm Thickness**	**1.50 cm Thickness**
900	6.158492	6.203884	6.254112
1200	6.111322	6.162867	6.222897
1500	6.050674	6.111596	6.184745
1800	5.976550	6.050070	6.139656
2100	5.888948	5.978290	6.087630
2400	5.787869	5.896255	6.028668
2700	5.673313	5.803967	5.962768
3000	5.545280	5.698006	5.889932
3300	5.403770	5.592045	5.813628
3600	5.248782	5.475829	5.733855

**Table 3b:** Exergy outflow (kJ/s) at 75 °C.

**Time(s)**	**0.50 cm Thickness**	**1.00 cm Thickness**	**1.50 cm Thickness**
900	9.155206	9.309953	9.456342
1200	9.049277	9.214883	9.378876
1500	8.914457	9.094794	9.280752
1800	8.750747	8.949687	9.192957
2100	8.558147	8.779561	9.053517
2400	8.336657	8.584417	8.893420
2700	8.086278	8.364255	8.717830
3000	7.807008	8.119074	8.511253
3300	7.498848	7.848874	8.289183
3600	7.161799	7.553656	8.046455

**Table 3c:** Exergy outflow (kJ/s) at 85 °C.

**Time(s)**	**0.50 cm Thickness**	**1.00 cm Thickness**	**1.50 cm Thickness**
900	12.41311	12.88182	13.17980
1200	12.20396	12.67903	13.00232
1500	11.94560	12.42893	12.78224
1800	11.63803	12.17206	12.56216
2100	11.28125	11.82732	12.25689
2400	10.87525	11.43526	11.90902
2700	10.42005	11.00265	11.52566
3000	9.915628	10.50920	11.08550
3300	9.638813	10.27937	10.92932
3600	9.361998	10.05630	10.78733

**Table 3d:** Exergy outflow (kJ/s) at 95 °C.

**Time(s)**	**0.50 cm Thickness**	**1.00 cm Thickness**	**1.50 cm Thickness**
900	15.96692	16.57502	17.06327
1200	15.59396	16.20410	16.71951
1500	15.13149	15.74252	16.28762
1800	14.57952	15.19026	15.76758
2100	13.93803	14.54734	15.15940
2400	13.20704	13.81375	14.46308
2700	12.83408	13.32084	13.67862
3000	12.50588	13.07192	13.45827
3300	12.26719	12.71748	13.25554
3600	12.02850	12.53615	13.13214

**Table 4a:** Exergy loss (kJ/s) at 65 °C.

**Time(s)**	**0.50 cm Thickness**	**1.00 cm Thickness**	**1.50 cm Thickness**
900	0.160008	0.114616	0.064388
1200	0.207178	0.155633	0.095603
1500	0.267826	0.206904	0.133755
1800	0.341950	0.268430	0.178844
2100	0.429552	0.340210	0.230870
2400	0.530631	0.422245	0.289832
2700	0.645187	0.514533	0.355732
3000	0.773220	0.620494	0.428568
3300	0.914730	0.726455	0.504872
3600	1.069718	0.842671	0.584645

**Table 4b:** Exergy loss (kJ/s) at 75 °C.

**Time(s)**	**0.50 cm Thickness**	**1.00 cm Thickness**	**1.50 cm Thickness**
900	0.522894	0.368147	0.221758
1200	0.628823	0.463217	0.299224
1500	0.763643	0.583306	0.397348
1800	0.927353	0.728413	0.485143
2100	1.119953	0.898539	0.624583
2400	1.341443	1.093683	0.784680
2700	1.591822	1.313845	0.960270
3000	1.871092	1.559026	1.166847
3300	2.179252	1.829226	1.388917
3600	2.516301	2.124444	1.631645

**Table 4c:** Exergy loss (kJ/s) at 85 °C.

**Time(s)**	**0.50 cm Thickness**	**1.00 cm Thickness**	**1.50 cm Thickness**
900	1.26319	0.79448	0.49650
1200	1.47234	0.99727	0.67398
1500	1.73070	1.24737	0.89406
1800	2.03827	1.50424	1.11414
2100	2.39505	1.84898	1.41941
2400	2.80105	2.24104	1.76728
2700	3.25625	2.67365	2.15064
3000	3.76067	3.16710	2.5908
3300	4.03748	3.39693	2.74698
3600	4.31430	3.62000	2.88897

**Table 4d:** Exergy loss (kJ/s) at 95 °C.

**Time(s)**	**0.50 cm Thickness**	**1.00 cm Thickness**	**1.50 cm Thickness**
900	2.30918	1.70108	1.21283
1200	2.68214	2.07200	1.55659
1500	3.14461	2.53358	1.98848
1800	3.69658	3.08584	2.50852
2100	4.33807	3.72876	3.11670
2400	5.06906	4.46235	3.81302
2700	5.44202	4.95526	4.59748
3000	5.77022	5.20418	4.81783
3300	6.00891	5.55862	5.02056
3600	6.24760	5.73995	5.14396

## 2 Experimental design, materials and methods

The paramount objective of any drying process is the utilization of minimum amount of energy to obtain a maximum amount of moisture removal with a view to achieving the desired product conditions and specifications. Drying is a complex process of heat and mass transfer for removal of moisture from a wet solid. Two separate phenomena are involved in drying. One, moisture must travel from the interior of a material to the surface of that material either by capillary action or diffusion and two, evaporation of the surface water into the surrounding air [1].

Exergy is a parameter of the second law of thermodynamics and it is defined as the maximum work quantity which can be produced by a system from flow of matter, heat or energy when equilibrium is reached with the environment as reference **[**2**]**. Exergy is a combined property of a system and its environment because it depends on the state of both the system and environment. It is neither a thermodynamic property of matter nor a thermodynamic potential of a system and the exergy of a system in equilibrium with the environment is zero [3].

Exergy is conserved only during ideal processes and lost or destroyed in actual processes due to irreversibilities [4].

Exergy analyses is a reliable method to establish strategies to design, implement and operate many industrial processes in which optimal energy usage is sacrosanct with a view to obtaining relevant information pertaining to plant and operation costs, energy conservation, fuel versatility and pollutants level [5], [6].

Exergy analysis plays an important role in optimization of drying conditions and drying system performance improvement [7].

### 2.1 Experimental Procedure

Fresh samples of onion fruits were bought from local market in otta, Ogun state, Nigeria. The onions were washed with distilled water to remove particles and contaminants that can adversely affect the experimental results. Hence, 36.50 g of the sample at different thicknesses of 0.50 cm, 1.00 cm and 1.50 cm were taken into the cabinet dryer for drying at different temperatures of 65°C, 75 °C,85 °C and 95°C and the weight loss at each temperature and thickness was determined with the aid of a digital weighing balance.

### 2.2 Exergy Analyses

Exergy analyses are typically performed to determine the location, type and magnitude of thermodynamic inefficiencies during drying process by applying the second law of thermodynamics [8].

The reduced form of exergy equation is given by Eq. (1) below:

(1) $$\mathrm{Ex}=\mathrm{mc}\left[\left(T-T_{\infty }\right)-T_{\infty }\ln\left(T/T_{\infty }\right)\right]$$

Where:

Ex = Exergy (kJ/s)
C = specific heat [kJ/kgK]
$T_{\infty }$ = reference temperature (25 °C or 298 K)
T = drying air temperature (K)
m = mass flow rate of fresh or dried product.

The specific heat of the fresh and dried product $C_{p\left[\mathrm{KJ}/\mathrm{Kg}K\right]}$ was also calculated by using the Eq (2). (9) proposed by [9] as:

(2) $$c_{p}=4.187X_{m}\;+\;1.424X_{c}\;+\;1.549X_{p}\;+\;1.675X_{f}\;+\;0.837X_{a}$$

where $X_{m}$ = moisture component (%), $X_{c}$ = carbohydrate component (%), $X_{p}$ = protein component (%), $X_{f}$ = fat component (%) $X_{a}$ = ash component (%)

The exergy inflow represents the maximum amount of useful available energy that is being supplied into any system (e.g batch dryer) to cause a change in either the properties of the system or any material within the surroundings of the system.

Exergy inflow can be expressed by Eq. (3) below

(3) $${\mathrm{Ex}}_{\mathrm{in}}={\mathrm{Ex}}_{\mathrm{ain}}\;+\;{\mathrm{Ex}}_{\mathrm{FO}}$$

${\mathrm{Ex}}_{I}$ = exergy inflow (kJ/s),
${\mathrm{Ex}}_{\mathrm{ain}}$= exergy inflow of air (kJ/s) and
${\mathrm{Ex}}_{\mathrm{FO}}$ = exergy of fresh onion (kJ/s)

Similarly, Eq. (4) gives the general form of exergy outflow.

(4) $${\mathrm{Ex}}_{\mathrm{out}}={\mathrm{Ex}}_{a0\mathrm{ut}}\;+\;{\mathrm{Ex}}_{\mathrm{DO}}\;+\;{\mathrm{Ex}}_{\mathrm{ldc}}$$

${\mathrm{Ex}}_{\mathrm{out}}$ = exergy outflow (kJ/s)
${\mathrm{Ex}}_{a0\mathrm{ut}}$ = exergy outflow of air (kJ/s)
${\mathrm{Ex}}_{\mathrm{ldc}}$ = exergy destruction (kJ/s)

Since mass flow rate of drying air was evenly distributed throughout the whole cross section of drying chamber,

Hence, initial mass flow rate of air is equal to the final mass flow rate of air

(5) $$m_{\mathrm{ai}}=m_{\mathrm{ao}}$$

Thus,

(6) $${\mathrm{Ex}}_{\mathrm{ain}}=\;{\mathrm{Ex}}_{\mathrm{aout}}$$

The exergy destruction, that is, exergy loss resulting from heat loss through the drying chamber can be described by Eq. (7) [10], [11].

(7) $${\mathrm{Ex}}_{\mathrm{ldc}}=Q_{\mathrm{ldc}}[1-\frac{T_{\infty }}{T_{\mathrm{dc avg}}}]$$

Where $T_{\mathrm{dc avg}}$ is the average temperature of the drying chamber and $Q_{\mathrm{ldc}}$ is the heat loss by drying chamber which is assumed to be negligible. Hence, ${\mathrm{Ex}}_{\mathrm{ldc}}=0$

Exergy loss is an energy parameter that is often confused with exergy destruction. It represents the transfer of exergy from a system to its external environment in an irreversible manner (the discharge of a non-useful energy stream into the surroundings) while exergy destruction is an internal phenomenon that characterizes exergy destruction due to irreversibilities within a component of a system (e.g exergy destruction during combustion process).

Exergy loss was calculated by using Eq. (8),

(8) Exergyloss=Exergyinflow−Exergyoutflow

Exergy efficiency is a critical indicator of the quality level of the converted energy. The exergy efficiency of a system is maximized when exergy loss is minimized and it is mathematically represented by Eq. (9).

(9) $$\mathrm{Exergy}\;\mathrm{efficiency}=\frac{\mathrm{Exergy}\;\mathrm{out}\;\mathrm{flow}}{\mathrm{Exergy}\;\mathrm{inflow}}$$

It can also be expressed by Eq. (10)

(10) $$\mathrm{Exergy}\;\mathrm{efficiency}=1-\frac{\mathrm{Exergy}\;\mathrm{loss}}{\mathrm{Exergy}\;\mathrm{inflow}}$$

The exergetic sustainability index (ESI) is a dimensionless parameter that is based on the exergy analysis and it is defined as the relationship between the input exergy and exergy losses of a system. The parameter provides us with useful information about the process influence on the environment [12]. Improvement on exergy efficiency will naturally translate to higher sustainability index.

Mathematically, it is represented by Eq. (11).

(11) $$\mathrm{ESI}=\frac{1}{1-\mathrm{Exergy}\;\mathrm{efficiency}}$$

Exergy improvement potential measures are necessary to increase exergy efficiency with a view to reducing environmental impact by reducing energy losses [13]. lower exergy efficiency would lead to higher improvement potential [14], [15].

(12) EIP=Exergyloss*(1−Exergyefficiency)
